# Hypertensive emergency presenting with diffuse alveolar hemorrhaging and thrombotic microangiopathy: A case report and review of the literature 

**DOI:** 10.5414/CNCS109939

**Published:** 2020-07-27

**Authors:** Mayumi Ito, Takayuki Katsuno, Asako Kachi, Yasuhiko Ito

**Affiliations:** Department of Nephrology and Rheumatology, Aichi Medical University, Nagakute, Japan

**Keywords:** hypertensive emergency, diffuse alveolar hemorrhage, thrombotic microangiopathy

## Abstract

There are few studies reporting diffuse alveolar hemorrhage (DAH) caused by hypertensive emergency. We describe a 41-year-old man who visited the emergency room with hemoptysis and dyspnea. He had a 5-year history of hypertension, though he had not received any treatment. His blood pressure was 233/159 mmHg, his percutaneous oxygen saturation level was 88% on room air, and he had a serum creatinine level of 11.7 mg/dL. Laboratory data showed microangiopathic hemolytic anemia, thrombocytopenia, and severe kidney damage, suggesting thrombotic microangiopathy (TMA). Chest computed tomography and bronchoalveolar lavage revealed pulmonary alveolar hemorrhage. In addition to steroid treatment and plasma exchange, antihypertensive therapy was started immediately. On day 3, activity of a disintegrin and metalloproteinase with thrombospondin type 1 motif, member 13 (ADAMTS13) activity was not significantly reduced, and clinical markers for vasculitis and connective tissue disease were negative. Therefore, steroid administration and plasma exchange were discontinued. Although antihypertensive therapy centering on angiotensin II receptor blocker was effective for DAH and TMA, renal function did not recover, and maintenance hemodialysis was required. Renal pathological findings were consistent with malignant nephrosclerosis, and features suggestive of vasculitis were not found. The pathophysiology in this case was considered to be mainly hypertension and vascular endothelial injury with renin-angiotensin-aldosterone system (RAAS) activation. The use of RAAS inhibitor was effective in converging DAH and TMA, and it was expected to repair vascular endothelial damage associated with appropriate antihypertensive intervention. The authors present this rare condition with a review of previous reports.

## Introduction 

Hypertensive emergency (HE) is a clinical syndrome characterized by severe hypertension with progressive renal failure, heart failure, and bilateral retinal hemorrhages and/or exudates [1]. In HE, thrombotic microangiopathy (TMA) is an occasional complication, particularly in patients with kidney impairment [2]. It is believed that severe hypertension causes endothelial damage of arterioles and glomerular capillaries and progresses to TMA. On the other hand, HE is rarely associated with diffuse alveolar hemorrhage (DAH). Herein, we report a case of HE complicated by DAH and TMA presenting with severe renal failure. Generally, pulmonary renal syndrome, which features rapidly progressive renal dysfunction and DAH, is induced by an autoimmune-mediated mechanism [3]. To date, there have only been 6 reports of HE with DAH, and none have mentioned TMA. To the best of our knowledge, this is the first case report on DAH and TMA associated with HE. The authors discuss this case with a review of previous literature. 

## Case presentation 

A 41-year-old man visited a local doctor with a chief complaint of dyspnea and hemoptysis for 2 days. The patient was initially diagnosed with severe hypertension and renal failure and was then referred to our medical facility. He was diagnosed with hypertension 5 years prior but had not received any treatment. There was no recent history of infectious gastroenteritis or diarrhea. 

**Figure 1.**
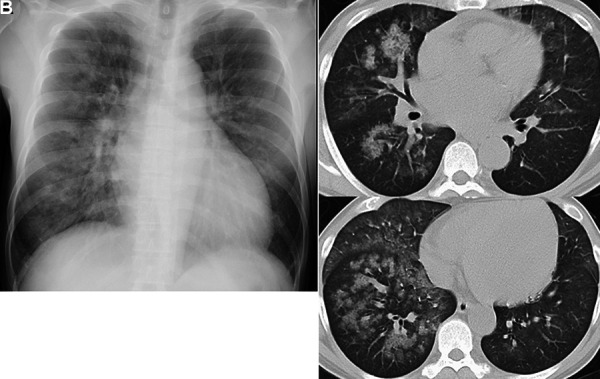
A: Chest radiograph showing extensive bilateral alveolar shadowing and cardiomegaly; cardiothoracic ratio 60%. B: High-resolution chest computed tomography on the first day revealed diffuse perihilar ground-glass attenuation with some areas of consolidation along the bronchial vascular bundle.

**Figure 2.**
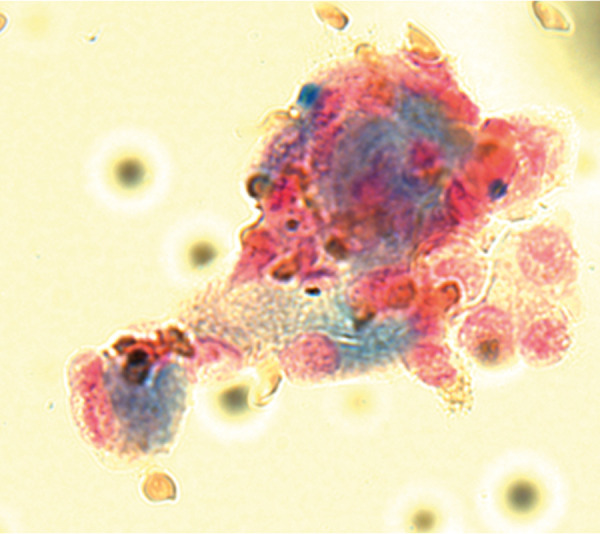
In bronchoalveolar lavage, a large number of hemosiderin-laden macrophages are histologically confirmed, which indicate an alveolar hemorrhage (Berlin blue stain).

On admission, a physical examination showed his blood pressure was 233/159 mmHg, heart rate was 135 bpm, and body temperature was 37.7 °C. His percutaneous oxygen saturation level was 88% on room air, and coarse crackles were heard in both lower lung fields. The patient was alert and oriented. Jugular venous distention was observed. Swelling and tenderness of the joints, rash, lower extremity edema, and neurological abnormalities were not observed. There was no skin tightening. Fundoscopy indicated bilateral hemorrhages and cotton wool spots without papilledema, which corresponded to hypertensive retinopathy according to Keith-Wagener-Barker classification III. Clinical laboratory findings from day 1 are presented in Table 1. A chest radiograph represented extensive bilateral alveolar shadowing and cardiomegaly (cardiothoracic ratio: 60%). There were no pleural effusions (Figure 1A). High-resolution chest computed tomography (CT) showed diffuse perihilar ground-glass attenuation with some areas of consolidation along the bronchial vascular bundle (Figure 1B). Using a bronchoalveolar lavage fluid test, macroscopic alveolar hemorrhage was observed and a large number of hemosiderin-laden macrophages were histologically confirmed (Figure 2). Abdominal CT revealed no apparent atrophy in either kidney, a small amount of ascites and dilation of the inferior vena cava. An echocardiogram indicated eccentric left ventricular (LV) hypertrophy with systolic and diastolic dysfunctions; LV wall motion showed diffuse severe hypokinesis, and the ejection fraction value was 25.4%. Coronary angiography revealed no significant stenosis that would require therapeutic intervention. The patient was diagnosed with hypertensive heart failure. 

**Figure 3.**
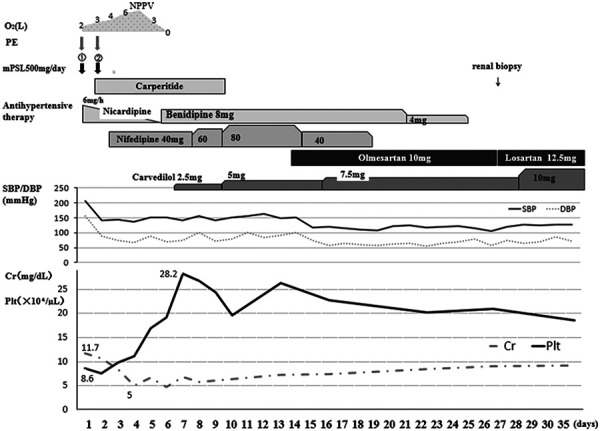
Clinical course in this case. PE = plasma exchange; mPSL = methylprednisolone; SBP = systolic blood pressure; DBP = diastolic blood pressure; NPPV = noninvasive positive pressure ventilation; Cr = creatinine; Plt = platelet count.

**Figure 4.**
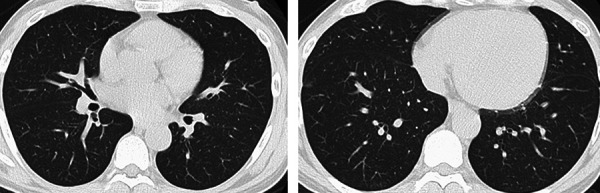
Alveolar bleeding gradually disappeared in the chest computed tomography on day 35.

According to the clinical findings, the patient was diagnosed with HE with pulmonary alveolar hemorrhage and TMA. Diagnosis of HE and TMA was made based on previous reports [4, 5]. The clinical course is shown in Figure 3. Since thrombotic thrombocytopenic purpura (TTP), vasculitis, and connective tissue disease could not be excluded at the time of admission, steroid administration and plasma exchange were started in addition to antihypertensive therapy with calcium antagonist (nicardipine) on day 1. Hemodialysis (HD) was also initiated on day 1 due to fluid overload. On day 3, various test results, including ADAMTS13 and autoantibodies, were identified and HE was considered to be the main pathophysiology. Therefore, steroid therapy and plasma exchange were discontinued. Although a β-blocker (carvedilol) was added to the calcium antagonist therapy on day 6, the patient’s blood pressure was 150 – 160/80 – 90 mmHg and the hypotensive effect was not enough. Consequently, an angiotensin II receptor blocker (olmesartan) was initiated in addition to these agents on day 14. As a result, hypertension improved promptly, and blood pressure management stabilized with losartan (12.5 mg/day) and carvedilol (10 mg/day). Alveolar hemorrhaging gradually improved, and the diffuse ground-glass shadows disappeared on chest CT images on day 35 (Figure 4). The platelet count increased to more than 100×10^3^/µL after day 5 and maintained at 150 to 200×10^3^/µL thereafter. Red blood cell fragments disappeared after day 2, and lactate dehydrogenase was normalized on day 23. However, renal function did not improve, and HD was continued. The first three HD sessions were performed for 3 hours and the fourth and subsequent sessions were performed for 4 hours with 1 – 3 L fluid removal per session because anuria was prolonged. A total of 14 HD sessions were performed at a frequency of 3 times per week during hospitalization. The patient’s peak body weight was 60.0 kg, which decreased to 54.0 kg after the final dialysis treatment prior to discharge. 

**Figure 5.**
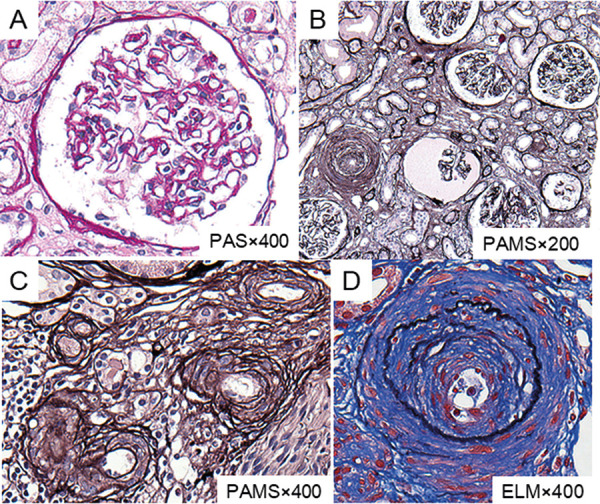
Light microscopic findings of kidney biopsy. A: In glomeruli, cell proliferative changes are scarce, and no findings suggestive of vasculitis, such as crescent formation, are observed (periodic acid-Schiff staining, original magnification × 400). B: The presented glomeruli show ischemic changes. The capillary walls are thickened and wrinkled. Tubular atrophy, dropout, and interstitial fibrosis are observed in a wide area (periodic acid-methenamine silver (PAMS) staining, original magnification × 200). C: Vascular lesions caused by persistent hypertension are characterized. In the arterioles, intimal thickening with proliferation of smooth muscle cells are observed (PAMS staining, original magnification × 400). D: Interlobular artery presents “onion skin” thickening with a narrowing lumen (Elastica masson staining, original magnification × 400).

Percutaneous renal biopsy was performed on day 27 to determine the cause of the kidney injury. Renal pathological images are presented in Figure 5. The sample contained 16 glomeruli, 4 of which showed global sclerosis. On light microscopy, the main glomerular lesions were ischemic changes, and the capillary walls were thickened and wrinkled. There was no significant mesangial proliferation, endocapillary hypercellularity, or extracapillary proliferation. Interstitial architecture showed advanced interstitial fibrosis and tubular atrophy. In the interlobular artery, multiple layers of elastic lamina were observed and some showed onion skin lesions. In addition, hyperplasia of medial smooth muscle cells, including the arcuate artery, was remarkable. Severe vascular endothelial damage, including endothelial cell swelling and edematous change, led to a narrowing and occlusion of the vascular lumen. Hyalinization was observed in the arterioles. Endothelial injury was evident in vessels of a size larger than the arteriole but was not observed in the glomerular capillaries. There were no findings of vasculitis or fibroid necrosis in the tissue. Immunofluorescent staining only revealed nonspecific immunoglobulin (Ig) G deposition on glomerular capillary walls, and IgA, IgM, complement component (C) 1q, C3, C4, and C4d were negative. Based on these pathological features, the patient was finally diagnosed with malignant nephrosclerosis. 

## Discussion 

This is the first case reporting a combination of DAH and TMA in HE. Activation of the renin-angiotensin-aldosterone system (RAAS) exists in the background of the pathological condition, and the RAAS inhibitor was effective for DAH and TMA. 

We searched previous case reports on HE with DAH and compared clinical findings, renal pathological features, and prognoses. To the best of our knowledge, there have been only 6 reports of HE manifesting with DAH [1, 6, 7, 8, 9, 10]. An overview of previous reports is shown in Table 2. All 6 cases were men, and the average age was 35.6 ± 8.1 years. Mean values of serum creatinine and blood pressure levels at the first visit were 6.3 ± 3.1 mg/dL and 224.7 ± 30.7 /134.9 ± 18.9 mmHg, respectively. Our patient was also a man with a blood pressure level similar to the other cases, but the degree of renal failure was more severe. In renal pathological features, all cases presented consistent findings with malignant nephrosclerosis. The findings of our case were also comparable. Regarding treatment, blood pressure control was performed in all cases, and the details of antihypertensive treatment were described in 4 cases. Calcium antagonist and RAAS inhibitor were administered in all 4 cases, and a β-blocker was used in 3 cases. Steroid therapy was carried out in 2 cases, but treatment was discontinued within a few days. There was 1 case where cyclophosphamide was prescribed. In 5 cases, description of the clinical prognosis was recognized. DAH improved in all 5 cases, and renal function recovered in 4 cases. HD was performed in 2 cases, but only 1 case was transferred for maintenance HD. Since DAH was excluded due to an immunological mechanism, corticosteroid therapy was ceased for a short period of time and antihypertensive therapy was primarily performed in our case. As a result, DAH improved; however, renal function did not recover and maintenance HD was required. The main cause of irreversible renal failure in this case may have been that the patient already had advanced tubulointerstitial and vascular damage. 

It is also suggested that the merger of TMA may be one of the factors for the poor renal prognosis. TMA is a disease characterized by consumption thrombocytopenia and microangiopathic hemolytic anemia (MAHA), as well as organ damage associated with thrombotic pathologies. Approximately 27 – 43% [11, 12] of HE is complicated with TMA and, in these cases, kidney injury is more severe. Van den Born et al. [11] described that dialysis was needed in 58% of cases with concurrent TMA, but only in 3% of cases without TMA. RAAS activation and the subsequent endothelial damage play a central role in the pathogenesis of TMA in HE [13]. In HE, pressure diuresis causes fluid volume reduction, resulting in the activation of RAAS. Shear stress and activated RAAS induce endothelial cell injury, which leads to vascular hyperpermeability, inflammatory cytokine secretion, and the activation of adhesion molecules. As a result, intravascular platelet aggregation, thrombus formation, lumen narrowing, and fibrinoid necrosis occur, and TMA characterized by MAHA develops [3, 13]. In addition, the narrowing of interlobular and arteriole lumens caused by vascular endothelial injury induces a decrease in glomerular perfusion, creating a vicious circle in which further activation of RAAS promotes renal failure and hypertension. Srivastava et al. [14] suggested that damage to the pulmonary vascular endothelium may be the common pathophysiological event in DAH-complicated microangiopathic disease. In pulmonary arterial hypertension, which is mainly caused by vascular endothelial damage, it has been reported that local RAAS activity of the pulmonary artery and angiotensin-converting enzyme activity of pulmonary endothelial cells are increased [15]. In this case, severe RAAS activation occurred as indicated by plasma renin activity and plasma aldosterone concentration values. Despite severe renal failure, the presence of hypokalemia was consistent with RASS activation. Arterial blood gas analysis showed a mixture of metabolic acidosis related to severe renal dysfunction and metabolic alkalosis due to RAAS activation. It has been suggested that the administration of an angiotensin II receptor blocker interrupts the vicious circle described, attenuates pulmonary vascular endothelial injury through local RAAS suppression in the lungs, and may improve the pathology of DAH and TMA. 

TMA is classified into TTP, hemolytic uremic syndrome (HUS), complement-related HUS (atypical HUS (aHUS)), and secondary TMA having underlying diseases [16]. In this case, there was no significant decrease in ADAMTS13 activity and gastrointestinal symptoms, such as diarrhea. For these reasons, TTP and HUS were denied. However, it is often difficult to differentiate between hypertension-related TMA and aHUS. Complement abnormalities have been found and reported in patients with hypertension-related TMA [17]. It is assumed that in patients with genetic abnormalities related to complement regulatory factors, secondary factors – such as hypertension – trigger aHUS. Regarding our case, complement-related gene workup was not conducted. There were several reasons why aHUS is not strongly suggested. DAH and TMA improve with antihypertensive therapy. If DAH or thrombocytopenia are clinical symptoms caused by aHUS, refractory courses are expected. Renal pathological findings showed no evidence of endothelial injury or microthrombus in the glomeruli as well as negative immunostaining of the complement system, including C4d. There were few findings indicating complement activation, which was different from renal pathological features in aHUS. Furthermore, the patient did not have a characteristic family history suspecting aHUS. However, it is impossible to completely exclude aHUS from these points. In the future, if a new TMA-related organ disorder occurs, or before renal transplantation, genetic testing related to complement regulatory factors will be required. 

We presented a case of DAH and TMA associated with HE. HE should be included in the differential as a cause of alveolar hemorrhage. The pathological condition in this case was assumed to be mainly hypertension and vascular endothelial injury caused by RAAS activation. Treatment with RAAS inhibitor was effective in converging DAH and TMA, and it was expected to repair endothelial damage associated with antihypertensive intervention. However, the problem of poor renal prognosis was also indicated. It is necessary to accumulate and investigate cases in the future to clarify the pathophysiology and establish treatments for renal prognosis improvement. 

## Acknowledgment 

The authors appreciate the excellent technical assistance of M. Yamauchi, Y. Sugiyama, and Y. Watase. 

## Funding 

The authors have not received any external funding. 

## Conflict of interest 

The authors have no conflicts of interest to declare. 

**Table 1.** Clinical laboratory findings at the time of admission to our facility.

Hematological tests	
White blood cell (µL)	9.9×10^3^
Red blood cell (µL)	275×10^4^
Hemoglobin (g/dL)	8.8
Platelet (µL)	86×10^3^
Reticulocyte count (µL)	13.4×10^4^
Red blood cell fragments (RBC)	3/1,000
Laboratory investigation	
Total serum protein (g/dL)	6.4
Albumin (g/dL)	4.0
Urea nitrogen (mg/dL)	84.6
Creatinine (mg/dL)	11.7
Estimated glomerular filtration rate (mL/min/1.73^2^)	5
Uric acid (g/dL)	11.6
Sodium (mmol/L)	138
Potassium (mmol/L)	2.6
Chloride (mmol/L)	98
Corrected calcium (mg/dL)	8.7
Phosphorus (mg/dL)	5.0
Total cholesterol (mg/dL)	242
Low-density lipoprotein cholesterol (mg/dL)	156
Aspartate aminotransferase (U/L)	16
Alanine aminotransferase (U/L)	9
γ-glutamyl transpeptidase (U/L)	9
Lactate dehydrogenase (U/L)	749
Alkaline phosphatase (U/L)	124
Total bilirubin (mg/dL)	2.51
Direct bilirubin (mg/dL)	0.29
Glucose (mg/dL)	119
Hemoglobin A1c (%)	4.0
C-reactive protein (mg/dL)	1.42
Brain natriuretic peptide (pg/mL)	2,277
Ferritin (ng/mL)	354.9
Haptoglobin (mg/dL)	< 10
ADAMTS13 activity (%)	67
Adrenalin (pg/mL)	236
Noradrenaline (pg/mL)	1,968
Plasma aldosterone concentration (pg/mL)	799
Plasma renin activity (ng/mL/h)	≥ 20
Immunological study	
Immunoglobulin G (mg/dL)	789
Immunoglobulin A (mg/dL)	254
Immunoglobulin M (mg/dL)	48
Complement component 3 (mg/dL)	78
Complement component 4 (mg/dL)	28.5
Total hemolytic complement (mg/dL)	55.5
Antinuclear antibody	< 40
Rheumatoid factor (IU/mL)	< 5.0
Proteinase 3-anti neutrophil cytoplasmic antibody (U/mL)	< 1.0
Myeloperoxidase-anti neutrophil cytoplasmic antibody (U/mL)	< 1.0
Anti-glomerular basement membrane antibody (U/mL)	< 2 .0
Anti-topoisomerase I antibody	Negative
Anti-centromere antibody	Negative
Anti-RNA polymerase III antibody	Negative
Direct Coombs test	Negative
Indirect Coombs test	Negative
Infection related survey	
Hepatitis B surface antigen	Negative
Hepatitis B surface antibody	Negative
Hepatitis B core antibody	Negative
Hepatitis C antibody	Negative
Human immunodeficiency virus antibody	Negative
Coagulation tests	
Prothrombin time-international normalized ratio	1.22
Activated partial thromboplastin time (s)	29.3
Control (s)	27.4
Fibrinogen (mg/dL)	487
Fibrinogen degradation product (µg/mL)	4.10
D-dimer (µg/mL)	0.91
Arterial blood gas analysis (O_2_ 2 L×min)	
pH	7.497
Partial pressure of oxygen (mmHg)	80.8
Partial carbon dioxide pressure (mmHg)	27.8
Bicarbonate (mmol/L)	20.9
Base excess (mmol/L)	–1.5
Urinalysis	
pH	5.5
Protein (0.17g/24h)	2+
Blood (1 – 4 RBCs/HPF)	1+
Glucose	–
White blood cell (per HPF)	< 1
β_2_ macroglobulin (µg/L)	4,190
N-acetylglucosamine (U/L)	13.3
Granular casts (per WF)	20 – 29
Waxy casts (per WF)	5 – 9

ADAMTS13 = a disintegrin and metalloproteinase with thrombospondin type 1 motif, member 13; HPF = high-power field; WF = whole field.

**Table 2.** A review of previously reported cases of malignant hypertension with diffuse alveolar hemorrhage and comparison with this case.

Age	Sex	Symptom	blood pressure (mmHg)	sCr (mg/dL)	Renal pathology	Treatment	Outcome	References
34	Male	Hemoptysis, dyspnea, headache, blurred vision	220/135	4.9	Fibrinoid necrosis of the afferent arterioles, proliferative endoarteritis at the interlobular arteries	CA, ACE-I, hemodialysis, prednisolone cyclophosphamide	Renal function was gradually recovered, and pulmonary hemorrhage completely disappeared by treatment with antihypertensive agents.	[6]
26	Male	Hemoptysis, exertional dyspnea	210/150	3.1	The capillary walls were thickened and wrinkled. A small artery showed “onion peel” thickening with a narrowed lumen.	CA, β-blocker, ARB, artificial breathing management	N/A	[7]
38	Male	Blurring of vision, hemoptysis, dyspnea	220/120	4.43	Ischemic collapse of glomerulus, severe fibrointimal thickening of the arteries with fibrinoid deposits in the wall, medial hypertrophy and hyaline arteriosclerosis of arteries	CA, β-blocker, ACE-I, nitro-glycerine infusion	Chest X-ray returned to normal 4 weeks later. Not require dialysis. 18 months on, serum creatinine is stable at 3.15 mg/dL with good blood pressure control.	[8]
32	Male	Hemoptysis, general fatigue	290/150	9	Fibrinoid necrosis in the afferent arterioles, Onion skin appearance in the interlobular arteries, Some glomeruli have collapse, Tubular atrophy and interstitial fibrosis	CA, β-blocker, ACE-I	Not require dialysis, The normalization of blood pressure allowed serum creatinine level decreased to 6.7 mg/dL on the 22nd day. The infiltrating shadows of the chest CT disappeared.	[1]
51	Male	Dry cough, orthopnea	220/130	8.02	Histopathological characteristics of hypertensive nephrosclerosis	Anti-hypertensive drugs and Hemodialysis	Maintenance dialysis. Lung opacities started to clear within two days of presentation.	[10]
27	Male	Hemoptysis	180/100	3.11	Smooth muscle cell hyperplasia in the media of interlobular artery, Fibrinoid necrosis and intramural thrombi of small arterioles, collapsed glomerulus by ischemia	Steroid pulse therapy (discontinued in a few days), blood pressure control	Not require dialysis, Serum creatinine level was 1.98 mg/dL at 10 months after discharge.	[9]
41	Male	Cough, hemoptysis, dyspnea	233/159	11.69	Collapsed glomerulus by ischemia A small artery showed onion skin thickening with a narrowed lumen. Tubular atrophy and interstitial fibrosis	Steroid pulse therapy, plasma exchange (discontinued in a few days),CA, ARB, β-blocker	Maintenance dialysis, Pulmonary hemorrhage completely disappeared, and TMA pathology improved promptly by treatment with antihypertensive agents.	This case

sCr = serum creatinine; CA = calcium antagonist; ACE-I = angiotensin-converting-enzyme inhibitor; ARB = angiotensin II receptor blocker; N/A = not applicable; TMA = thrombotic microangiopathy.
